# A Literature Review of Women's Sex Hormone Changes by Acupuncture Treatment: Analysis of Human and Animal Studies

**DOI:** 10.1155/2018/3752723

**Published:** 2018-11-15

**Authors:** Jade Heejae Ko, Seung-Nam Kim

**Affiliations:** College of Korean Medicine, Dongguk University, Goyang 10326, Republic of Korea

## Abstract

**Background:**

It has been known that acupuncture treatment relieves gynecological disorders such as menopause, ovarian dysfunction, and dysmenorrhea. Sex hormones, including estrogen, progesterone, and gonadotropins, are related to the women disease. However, regulative effect of acupuncture on sex hormones has not been fully identified.

**Methods:**

Acupuncture articles including analysis of sex hormones were searched in electronic databases from inception to June 2018. The methodological quality was assessed using modified CAMRADES tool. A total of 23 articles were selected and analyzed.

**Results:**

In the results, overall studies showed that acupuncture increases estrogen, especially estradiol, progesterone, prolactin, and other hormones. Estradiol level was increased in most of studies except 3 studies which resulted in decreased level or not meaningful change. Two studies showed increase of FSH and LH whereas it was decreased in other studies. Other hormones were mostly increased by acupuncture.

**Conclusion:**

This study possibly indicates that acupuncture changes sex hormone in various gynecological conditions in women.

## 1. Background

Female reproductive health is closely related to menstrual cycle and menstrual health. The causes of gynecological disorders, reproductive dysfunctions, and menopausal syndromes such as polycystic ovarian syndrome (PCOS), dysmenorrhea, and hot flush were suggested in numerous studies. The cause of the gynecological issues is mainly imbalance of sex hormones [1–4] in addition to extrinsic factors [5]. Along with the scientific findings in terms of the dysfunctions and symptoms, various treatment methods have been used in patients. Hormonal contraceptives have been used most widely and frequently for gynecological disorders and reproductive dysfunctions [6]. For instance, oral contraceptives pills (OCPs), which are known to regulate hyperandrogenism, have been used to treat patients with PCOS and menstrual dysfunctions [7]. The efficacy and safety of the OCPs were proven via numerous experiments and clinical trials. Nevertheless, a considerable proportion of women who have ever taken OCPs discontinued taking OCPs due to side effects such as weight gain, headache, and nausea [8].

For gynecological issues of women, acupuncture has become one of the popular complementary treatment methods and use of acupuncture has been steadily increasing [9]. It has been well understood that acupuncture is effective in analgesia and blood flow regulation [10]. According to research, women are more likely to use acupuncture as an adjunct treatment compared to men [11, 12]. With increased recognition of effect of acupuncture in many field of studies, its effects on gynecological and reproductive issues were raised among people. Therefore, in present review, we explored how acupuncture affects and changes different sex hormone levels in animal model and human.

## 2. Methods

### 2.1. Eligibility Criteria

All eligible studies examined changes in follicle stimulating hormone (FSH), luteinizing hormone (LH), FSH/LH, progesterone, estrogen, prolactin, or oxytocin level. Acupuncture treatment involving hormone studies were retrieved. Studies that only used either electroacupuncture or manual acupuncture were enrolled, while studies which used other acupoint stimulation method or moxibustion were excluded.

### 2.2. Study Selection

The following Pubmed, EMBASE, and Cochrane Central Register of Controlled Trials (CENTRAL) were searched from inception until June 2018. The search terms included the following: ([acupuncture OR electroacupuncture] AND [follicle stimulating hormone OR luteinizing hormone OR progesterone OR estrogen OR prolactin OR oxytocin OR reproductive hormone]). The searching of the electronic databases led us to identify 124 potentially relevant manuscripts. The titles and abstracts that met the criteria of our study were independently read by two reviewers and only complete manuscripts published in English were retrieved. A total of 101 articles were excluded and the exclusion criteria were as follows: (1) full texts not accessible, (2) review article, (3) use of other acupoint stimulation or moxibustion, (4) case report, (5) letter, (6) irrelevance in terms of hormone study, and (7) irrelevance in terms of acupuncture study. 23 studies were included ultimately.

### 2.3. Quality Assessment 

The methodological quality of each included study was assessed by two authors (Kim and Ko) by using 8-item checklist modified from CAMARADES checklist [13, 14]: (1) peer-reviewed manuscript, (2) explanation of acupuncture procedure, (3) detailed description of condition, (4) detailed statement of sample, (5) detailed explanation of sampling method, (6) statement of screened hormone type, (7) compliance with experiment subject welfare regulation, and (8) statement of potential conflict of interests. A sum of quality score was recorded for each article and possible total score was 8 points.

## 3. Results

### 3.1. Search Result

**Figure 1:**
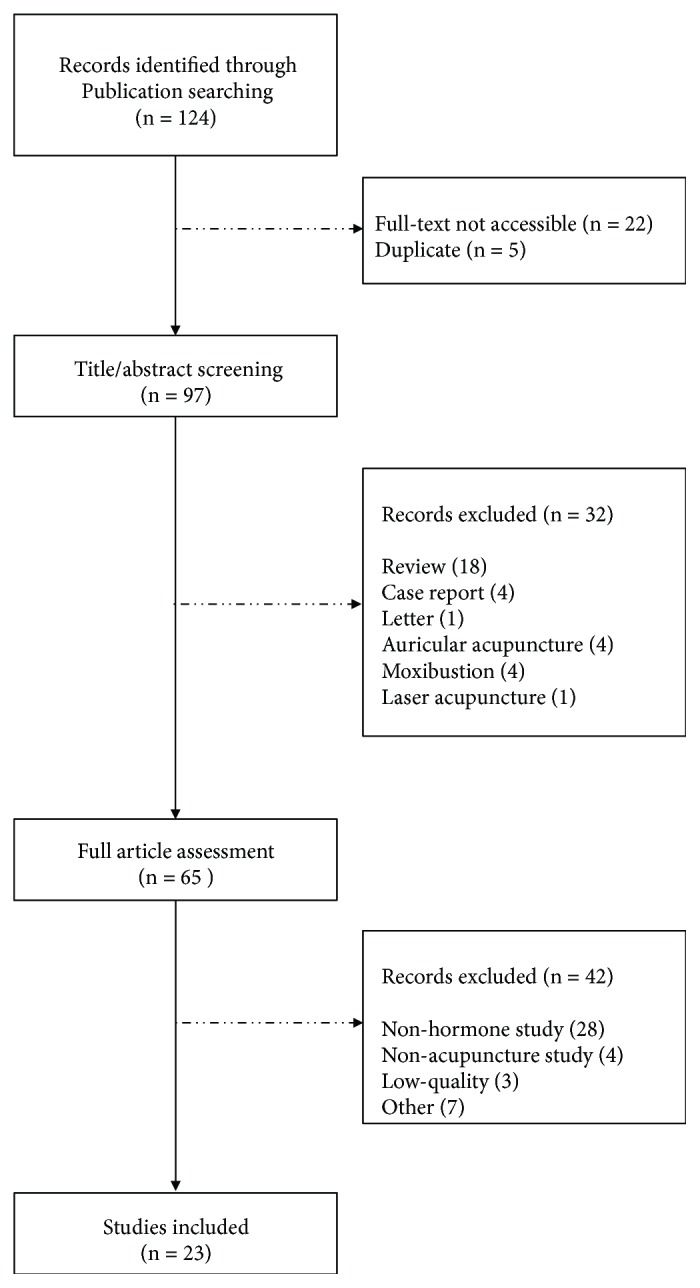
Flow diagram of the review.

A total of 124 publications were initially identified in Pubmed, EMBASE, and Cochrane Central Register of Controlled Trials (CENTRAL) from their inception to June 2018. Among the initial 124 publications, 22 articles were excluded because their full-texts were unobtainable, and 5 duplicate records were also excluded. After title and abstract screening, total of 32 articles were excluded because they were reviews, case reports, letters, or other acupoint stimulation methods. 42 articles were excluded additionally after full-text screening and there were 28 nonhormone studies, 4 nonacupuncture, 3 low-quality studies, and 7 studies which had other reasons. Among the 7 studies, 2 studies used animals other than rat or mouse, 1 study used herb as treatment, 1 study was conducted with male patients, and the remaining three studies induced diseases by estrogen or progesterone. 23 articles which satisfied the criteria of our study were ultimately selected. Our screening process is summarized as flow diagram in Figure 1.

13 articles used electroacupuncture (EA), 7 articles used manual acupuncture (MA), 2 articles used both EA and MA, and acupuncture method of one article was not able to identify. Of the 23 articles, 11 articles used human as subject and 12 articles used animal subject.

### 3.2. Quality Assessment

Table 1 shows quality assessment of included studies. The quality score of included studies ranged from 4 to 8 out of a total 8 points. The overall score for the studies were high. 9 studies scored 8 points [15–23], 10 studies scored 7 points [24–33], 2 studies scored 6 points [34, 35], and 2 studies scored 5 points [36, 37]. Of the 23 studies, 21 studies were peer-reviewed, 21 studies included explanation of acupuncture procedures, 22 studies described condition of experiment subject, and all 23 studies included detailed explanation of sample, sampling method, and screened hormone type. 22 studies mentioned compliance with experiment subject welfare regulations. 9 studies included potential conflicts of interest.

### 3.3. Effect of Acupuncture on Estrogen Level

We observed changes of estrogen level by acupuncture treatment of the 23 included studies (Table 2). Of the 23 studies, 14 studies investigated how acupuncture treatment affects changes in estrogen level solely or in addition to other female sex hormones. 3 articles measured estradiol (E2) level change in polycystic ovary syndrome (PCOS) models [15, 16, 24]. E2 level was decreased in 2 studies [15, 24] while there was no meaningful change in one study [16]. E2 level was increased in 5 ovariectomized rat model studies [17, 18, 27–29]. There were 3 articles which measured E2 level in human with menopausal syndrome [19, 34, 35]. There was no meaningful change in estrogen level in two of the studies [34, 35], while one study showed increment of E2 level [19].

Wang* et al.* and Zhou* et al.* investigated effect of acupuncture on E2 level in human with diminished ovarian reserve and primary ovarian insufficiency, respectively [20, 22]. E2 level was increased in compliance with acupuncture treatment in the both studies.

### 3.4. Effect of Acupuncture on FSH and LH Level

Table 3 shows 14 studies that examined effect of acupuncture and reported on the outcomes of FSH and LH level changes. Among the 14 studies, 6 studies measured FSH level, LH level, or FSH/LH level in PCOS models [15, 16, 23–26]. Maliqueo* et al.* suggested that acupuncture rendered FSH level increase, whereas LH level and FSH/LH level were decreased [23, 24]. Pastore* et al.* also found that FSH/LH level was upregulated with acupuncture treatment although there was no notable change in FSH level and LH level [16]. Changes in LH level in most of the studies were found to be decreased or not significant.

### 3.5. Effect of Acupuncture on Other Hormones

In Table 4, 10 studies suggested effect of acupuncture on hormonal changes by measuring several other sex hormones: progesterone [21, 25, 35], prolactin [21, 26, 35, 36], human chorionic gonadotropin (hCG) [16, 36], gonadotropin releasing hormone (GnRH) [29], oxytocin [31, 37], and prolactin releasing protein (PrRP) [27]. Each hormone was examined solely or along with FSH, LH, or estrogen. 3 studies investigated progesterone level in different pathological conditions and there was increment of progesterone level in the three studies [21, 25, 35]. Furthermore, other studies which examined prolactin, hCG, GnRH, oxytocin, and brain PrRP also suggested increased hormone level in compliance with acupuncture treatment.

## 4. Discussion

This review found that acupuncture potentially alters different sex hormone levels in animal model and patients with gynecological or reproductive issues. It is evident that acupuncture has been increasingly used in many diseases and its effects are examined through studies in various fields. Knowing that female population is high in number among acupuncture users, aim of understanding clinical significance and effect of acupuncture on women's sex hormone has been raised. We examined change in women's sex hormones: estrogen, progesterone, oxytocin, and gonadotropin hormones and searched evidence of hormones being regulated by acupuncture. A total of 23 studies were included for the review and hormone level change was investigated for each included study.

Based on what we have found, estrogen (estradiol) level was a mainly measured hormone in the articles and the increased estrogen was detected in most of the studies, while estrogen was decreased in the study of Maliqueo* et al.* [23, 24] and Johansson* et al.* [15]. As these two articles studied PCOS disease whereas other articles studied ovarian dysfunction, it is possible that increased estrogen level in ovarian dysfunction and decreased estrogen level in PCOS are presumably influenced by disease characteristics.

Gonadotropin hormone level was also investigated in most of the studies to see whether acupuncture affected hormone levels in animal model and patients. In an early hormonal study, Patel* et al.* suggested that GnRH-stimulated, oversecretion of LH best elucidates cause of PCOS in women [38]. Results were inconsistent among the studies; therefore further studies are expected for better understanding of effect of acupuncture in terms of gonadotropins.

Although there were comparatively less studies which examined other sex hormones, progesterone, oxytocin, hCG, PrRP, prolactin, and GnRH, hormone level was increased as a result of acupuncture. Among the studies, prolactin level was found to be increased by acupuncture. In several other studies, prolactin is increased as a result of acupuncture treatment [39, 40]. It seems that acupuncture potentially increases prolactin level, yet more mechanism studies are needed in order to verify exact effect of acupuncture on sex hormones.

In terms of acupuncture method, there was insufficient number of correlation identified between acupuncture method and hormone changes. For instance, estrogen level was increased by use of MA in the study of Sunay* et al.* [19] and there was also increased estrogen level in the study of Wang* et al.* [20] by using EA. By contrast, LH level was decreased in the study of Ma* et al.* [17]. However, the studies included in this review not only have broad range of acupuncture method, but also have different acupuncture forms (manual or electroacupuncture), durations of treatment, acupuncture points, and intensities (see in Supplementary Table 1). Such diversity of acupuncture method made drawing definite conclusion hardly possible. Thus, further study is required to understand the relationship between sex hormones and acupuncture method. Meanwhile, effect of homeostasis regulating effect of acupuncture may elucidate mechanism in hormone changes. It was studied how acupoint specificity affects hypothalamic-pituitary-adrenal cortex axis (HPAA) regulation. In addition, it was suggested that acupuncture altered stress reaction neurons in paraventricular nucleus [41]. One of the key factors in brain activity and homeostasis regulation, Neuropeptide Y (NPY), which is highly concentrated in hypothalamus, is involved in the regulation of various physiological functions such as energy homeostasis and stress-related behavior process. Several studies investigated effect of acupuncture on critical physiological processes. In diabetic rats, acupuncture suppressed NPY and it led to controlling hyperphagia [42]. Anxiogenic-like behaviors in maternally separated rats were also recovered to normal state and decreased NPY expression was upregulated by acupuncture [43]. Acupuncture prevented elevation of NPY expression which was induced by cold stress [44]. The hormonal changes were inconsistent among same disease models. The inconsistency is considered to be due to different reasons of disease and used acupoints. It is suggested to focus more on the fact that acupuncture may restore the balance among the hormones rather than solely focusing on increase and decrease of hormone level. To suggest clinical implications of acupuncture in various diseases, it is important to recognize that acupuncture may have bidirectional effect depending on diseases. It also should be considered what physiological context a disease potentially has in order to properly account for effect of acupuncture.

## 5. Conclusion

Our study has shown that acupuncture altered some of the sex hormone levels in both animal model and human. It is difficult to conclude that this review provided strong evidence to elucidate regulative effect of acupuncture on sex hormones. Still, we believed that result of this review and holistic approach to effect of acupuncture on sex hormones will contribute to the field of hormonal study and acupuncture study as further guidance and reference.

## Figures and Tables

**Table 1:** Quality assessment of studies included.

Author	Year	Q1	Q2	Q3	Q4	Q5	Q6	Q7	Q8	Score
Maliqueo *et al.*	2015	✓	✓	✓	✓	✓	✓	✓		7
Johansson *et al.*	2013	✓	✓	✓	✓	✓	✓	✓	✓	8
Feng *et al.*	2012	✓	✓	✓	✓	✓	✓	✓		7
Pastore *et al.*	2011	✓	✓	✓	✓	✓	✓	✓	✓	8
Stener-Victorin *et al.*	2000	✓	✓	✓	✓	✓	✓	✓		7
Ma *et al.*	2017	✓	✓	✓	✓	✓	✓	✓	✓	8
Qin *et al.*	2013	✓	✓	✓	✓	✓	✓	✓	✓	8
Yao* et al.*	2007	✓	✓	✓	✓	✓	✓	✓		7
Zhao *et al.*	2004	✓	✓	✓	✓	✓	✓	✓		7
Zhao *et al.*	2003	✓	✓	✓	✓	✓	✓	✓		7
Sunay *et al.*	2011	✓	✓	✓	✓	✓	✓	✓	✓	8
Qu* et al.*	2007		✓	✓	✓	✓	✓	✓		6
Dong *et al.*	2001		✓	✓	✓	✓	✓	✓		6
Wang *et al.*	2016	✓	✓	✓	✓	✓	✓	✓	✓	8
Xiong *et al.*	2015	✓	✓	✓	✓	✓	✓	✓	✓	8
Zhou *et al.*	2013	✓	✓	✓	✓	✓	✓	✓	✓	8
Magarelli *et al.*	2009	✓		✓	✓	✓	✓			5
Zhang *et al.*	2007	✓	✓	✓	✓	✓	✓	✓		7
Gaudernack* et al.*	2006	✓	✓	✓	✓	✓	✓	✓		7
Yang *et al.*	2006	✓	✓	✓	✓	✓	✓	✓		7
Yang *et al.*	2006	✓	✓	✓	✓	✓	✓	✓		7
Uvnäs-Moberg *et al.*	1993	✓			✓	✓	✓	✓		5
Zheng *et al.*	2013	✓	✓	✓	✓	✓	✓	✓	✓	8

**Table 2:** Changes in estrogen by acupuncture treatment.

					**Changes in hormone level**		
**Author**	**Year**	**Subject**	**Condition**	**Number**	**Disease**	**Acupuncture**	**Acupuncture method**
				**of sample (exp/con)**		**compared to**	
						**disease**	
Maliqueo *et al.*	2015	Animal (rat)	PCOS	10/12	▲ E2	▽ E2	EA
Johansson *et al.*	2013	Human	PCOS	11/14		▽ E1, E1-S, E2	EA+MA
Feng *et al.*	2012	Animal (rat)	PCOS	40/44		▲ E2	EA+MA

Ma *et al.*	2017	Animal (rat)	Ovariectomized	8/8	▽ E2	▲ E2	EA
Qin *et al.*	2013	Animal (rat)	Ovariectomized	8/8		▲ E2	EA
Yao *et al.*	2007	Animal (rat)	Ovariectomized	8/8		▲ E2	EA
Zhao *et al.*	2004	Animal (rat)	Ovariectomized	10/12		▲ E2	EA
Zhao *et al.*	2003	Animal (rat)	Ovariectomized	10/12		▲ E2	EA

Sunay *et al.*	2011	Human	Menopausal syndrome	27/26	▽ E2	▲ E2	MA
Qu *et al.*	2007	Human	Menopausal syndrome	36/31		*≒* Estrogen	MA
Dong *et al.*	2001	Human	Menopausal syndrome	11		*≒* E2	MA

Wang *et al.*	2016	Human	Diminished ovarian reserve	21	▽ E2	▲ E2	EA

Zhou *et al.*	2013	Human	Primary ovarian insufficiency	11	▽ E2	▲ E2	EA

Zhang *et al.*	2007	Animal (rat)	Pubertal development	5/4	▽ E2	▲E2	EA

▲ Increased, ▽ decreased, and *≒* not significant

**Table 3:** Changes in FSH and LH by acupuncture treatment.

					**Changes in hormone level**		
**Author**	**Year**	**Subject**	**Condition**	**Number of sample (exp/con)**	**Disease**	**Acupuncture**	**Acupuncture method**
						**Compared to**	
						**disease**	
Maliqueo *et al.*	2015	Animal (rat)	PCOS	10/12	▽ FSH, ▲ LH	▲ FSH, ▽ LH, ▽ FSH/LH	EA
Johansson *et al.*	2013	Human	PCOS	11/14		*≒* FSH, *≒* LH, *≒* FSH/LH	EA+MA
Feng *et al.*	2012	Animal (rat)	PCOS	8/8		*≒* LH	EA+MA
Pastore *et al.*	2011	Human	PCOS	40/44		*≒* FSH, LH, ▲ FSH/LH	MA
Stener-Victorin *et al.*	2000	Human	PCOS	24		*≒* FSH, LH, ▽ FSH/LH	EA
Zheng *et al.*	2013	Human	PCOS	43		▽ FSH, ▽ LH, ▽ FSH/LH	MA

Ma *et al.*	2017	Animal (rat)	Ovariectomized	8/8	▲ LH	▽ LH	EA

Sunay *et al.*	2011	Human	Menopausal syndrome	27/26	▲ FSH, ▲ LH	*≒* FSH (*p* = 0.053), ▽ LH,	MA
Qu *et al.*	2007	Human	Menopausal syndrome	36/31		▽ FSH, *≒* LH	MA
Dong *et al.*	2001	Human	Menopausal syndrome	11		*≒* FSH (*p* = 0.04), *≒* LH	MA

Wang *et al.*	2016	Human	Diminished ovarian reserve	21	▲ FSH, ▲ LH	▽ FSH, ▽ LH, ▲FSH/LH	EA

Zhou *et al.*	2013	Human	Primary ovarian insufficiency	11	▲ FSH, ▲ LH	▽ FSH, ▽ LH	EA

▲ Increased, ▽ decreased, and *≒* not significant

**Table 4:** Changes in other hormones by acupuncture treatment.

					**Changes in hormone level**		
**Author**	**Year**	**Subject**	**Condition**	**Number of sample (exp/con)**	**Disease**	**Acupuncture compared to**	**Acupuncture method**
						**disease**	
Feng *et al.*	2012	Animal (rat)	PCOS	8/8	▽ Progesterone	▲ Progesterone	EA+MA
Pastore *et al.*	2011	Human	PCOS	40/44	▲ Testosterone	*≒* Testosterone	MA
Stener-Victorin *et al.*	2000	Human	PCOS	24	▽ Prolactin	▲ Prolactin	EA

Yao *et al.*	2007	Animal (rat)	Ovariectomized	8/8	▽ PrRP	▲ Brain PrRP	EA
Zhao *et al.*	2003	Animal (rat)	Ovariectomized	10/12	▽ GnRH	▲GnRH	EA

Dong *et al.*	2001	Human	Menopausal syndrome	11	▽ Progesterone, ▽ Prolactin	*≒* Progesterone, *≒* Prolactin	MA

Xiong *et al.*	2015	Animal (rat)	Embryo implantation dysfunction	6/6	▽ Progesterone, ▽ Prolactin	▲ Progesterone, ▲ Prolactin	MA

Magarelli *et al.*	2009	Human	*in vitro* fertilization	34/33	▽ hCG, ▽ Prolactin	▲ hCG, ▲ Prolactin	N/A

Gaudernack *et al.*	2006	Human	Labour	48/52	N/A	▽ need of oxytocin	MA

Uvnäs-Moberg *et al.*	1993	Animal (rat)	Normal	6/6	N/A	▲ oxytocin	EA

Yang *et al.*	2006	Animal (rat)	Analgesia	6/7	▽ oxytocin	*≒* oxytocin	EA
Yang *et al.*	2006	Animal (rat)	Analgesia	9/9	▽ oxytocin	*≒* oxytocin	EA

▲ Increased, ▽ decreased, *≒* not significant, and N/A; not applicable.

## Data Availability

The data sets supporting the conclusions of this article are included within the article.

## Supplementary Materials

Supplementary Materials Supplementary Table 1. Detailed description of acupuncture treatment in the studies.
