# Supplementary material for: A Literature Review of Women's Sex Hormone Changes by Acupuncture Treatment: Analysis of Human and Animal Studies
Source: Evid Based Complement Alternat Med. 2018 Nov 15;2018:3752723. doi: 10.1155/2018/3752723 (PMC6276442; doi:10.1155/2018/3752723)
Supplement: Supplementary Materials — Supplementary Table 1. Detailed description of acupuncture treatment in the studies. [file 3752723.f1.pdf]

Supplementary Table 1. Detailed description of acupuncture treatment in the studies

Author	Year	Acupuncture method	Subject	Point used	Duration	Detailed stimulation methods
Maliqueo *et al.*	2015	EA	Rat	N/A	15 min for 1st week, 20 min for 2nd, 3rd week, 25 min for 4th, 5th, and 6th week 30	2 Hz with 0.1 s, 80 Hz burst pulses, 0.6-1.4 mA
Johansson *et al.*	2013	EA+MA	Human	(CV3, CV6, ST29, SP6, SP9, LI4, GV20) OR (CV3, CV6, ST25, ST29, SP6, LR3, PC6, GV20)	min/day, twice/week for 10-13 weeks	2 Hz
Feng *et al.*	2012	EA+MA	Rat	N/A	15 min for 1st week, 20 min for 2nd, 3rd week, 25 min for 4th and 5th week	2 Hz with 0.1 s, 80 Hz burst pulses, 0.6-1.4 mA
Pastore *et al.*	2011	MA	Human	BL23, BL28, SP6, SP9.	Twice/ week for 4 weeks, once/week for 4 weeks	N/A
Stener-Victorin *et al.*	2000	EA	Human	N/A	twice/week for 4 weeks	2 Hz, 0.5 ms for 30 min
Ma *et al.*	2017	EA	Rat	CV3, CV4, SP6	30 min/day for 3 days	2 Hz, 0.6 ms, 0.8-1.4 mA
Qin *et al.*	2013	EA	Rat	BL18, BL20, BL23, ST36, SP6 CV3,	30 min/day for 14 days	10 Hz, 1-5 mA
Yao *et al.*	2007	EA	Rat	CV4, EX-CA1	30 min/day for 3 days	3 Hz, 0.2 ms, 80 pulses/s, 2mA
Zhao *et al.*	2004	EA	Rat	CV3, CV4, EX-CA1	30 min/day for 3 days	3 Hz, 0.2 ms, 80 pulses/s, 2mA
Zhao *et al.*	2003	EA	Rat	CV3, CV4, EX-CA1, SP6	30 min/day for 3 days	3 Hz, 2mA
Sunay *et al.*	2011	MA	Human	ST36, LI4, KI3, LR3, EX-HN3, CV3	20 min/day, twice/week,	without manual stimulation
Qu *et al.*	2007	MA	Human	SP6, GB20, CV4, LI4, KI3, EX-CA1	40 min/day, twice/week for 12 weeks	N/A
Dong *et al.*	2001	MA	Human	SP6, KI3, BL23	25 min/day, twice/week for 5 weeks	N/A
Wang *et al.*	2016	EA	Human	BL33 OR (CV4, ST25, EX-CA1)	20 min/day, 5 times/week for 4 weeks, 3 times/week for 12 weeks	dilatational wave, 0.5 ms, 2/15 Hz, 0.1-10mA
Xiong *et al.*	2015	MA	Rat	SP6, ST36	25 min/day, for 5-8 days	rotating and stirring needles at every 5 min
Zhou *et al.*	2013	EA	Human	BL33 OR (CV4, ST25, ST29)	20 min/day, 5 times/week for 4 weeks, 3 times/week for 2 months	continuous wave, 20 Hz, 1-4 mA
Magarelli *et al.*	2009	N/A	Human	P6, SP8, SP6, LV3, GV20, ST29, ST36, SP10, LI4	30 min/day, 11 times/ 24 hours before and 1 hour after ET	2 Hz, 0.5 ms for 30 min
Zhang *et al.*	2007	EA	Rat	GV20, GV4, BL23, KI3	30min/day for 10 days	single pulse, 30 Hz, 6 V

Gaudernack *et al.*	2006	MA	Human	ST36, LR3, CV4, TE6, LU7, HT7, LI4, GB41, KI3, KI6	20min/day approximately 24 h after PROM	N/A
Yang *et al.*	2006	EA	Rat	ST36	30 min/day	dense-disperse wave (10 Hz-20 Hz), 2-3 V
Yang *et al.*	2006	EA	Rat	ST36	30 min/day OR 10 min/day	dense-disperse wave (10 Hz-20 Hz), 2-3 V
Uvnäs-Moberg *et al.*	1993	EA	Rat	BL11, BL54	30 min/day	100 Hz
Zheng *et al.*	2013	MA	Human	CV4, CV6, CV10, CV12, ST21, ST25, ST28	30 min/day, twice/week for 6 months	N/A

N/A; not applicable
